# Surgical Embolectomy for a Clot-in-Transit Located in a Patent Foramen Ovale: A Case Report

**DOI:** 10.7759/cureus.81611

**Published:** 2025-04-02

**Authors:** Danielle Drew, Rayhan Karimi, Charles J Rousseau

**Affiliations:** 1 Clinical, Biomedical, and Educational Research, Edward Via College of Osteopathic Medicine, Spartanburg, USA; 2 Internal Medicine, Edward Via College of Osteopathic Medicine, Spartanburg, USA; 3 Cardiothoracic Surgery, Spartanburg Regional Medical Center, Spartanburg, USA

**Keywords:** iatrogenic shunt reversal, patent foramen ovale, pulmonary embolism, right heart dysfunction, surgical embolectomy, ventilation induction

## Abstract

Pulmonary embolism (PE) is a serious and potentially life-threatening medical condition that arises when a blood clot, typically originating in the deep veins of the legs or pelvis, travels through the bloodstream and lodges in the pulmonary arteries. This obstruction can impede blood flow to the lungs, leading to complications. PEs are a critical manifestation of venous thromboembolism, a condition characterized by the formation of blood clots within veins. The consequences of PE can vary, ranging from mild respiratory distress to severe respiratory failure or cardiac arrest, depending on the size and location of the clot. Prompt diagnosis and intervention are crucial to mitigate associated life-threatening implications. Patent foramen ovale (PFO) is an abnormality characterized by the persistence of a small opening between the atria of the heart. During fetal development, this opening, known as the foramen ovale, allows blood to bypass the non-functioning lungs. Normally, the foramen ovale closes shortly after birth. However, when it fails to seal completely, a PFO occurs. It is a relatively common variation and is often asymptomatic. However, it has gained attention in the medical field due to its association with certain health issues, particularly paradoxical embolism. This occurs when a blood clot, typically formed in the venous system, passes through the PFO and travels to the arterial circulation, potentially causing complications such as a stroke. This case report adds to the literature and highlights the importance of recognizing concurrent PFO and PE. A 30-year-old female patient arrived at the emergency room experiencing sudden difficulty breathing and diaphoresis. The presenting vital signs demonstrated mild sinus tachycardia and tachypnea with an oxygen saturation of 95% on room air. A transesophageal echocardiogram (TEE) was performed, which confirmed a mass extending into the septum and crossing the PFO. Catheter-directed thrombectomy was deemed unsafe and the patient was transferred emergently to the operating room for sternotomy, pulmonary embolectomy, extraction of PE-in-transit, and PFO closure. Upon administration of general anesthesia, the patient experienced a significant decrease in blood pressure and oxygen saturation. A repeat TEE was performed and it revealed profound right heart dysfunction with an absence of the PE that had previously been lodged in the interatrial septum. Urgent bilateral pulmonary embolectomy was undertaken to extract the embolism in transit from the pulmonary artery. This case report highlights a PE-in-transit at the interatrial septum through a PFO following induction of positive pressure ventilation and anesthesia, resulting in a hemodynamic collapse. The benefits of surgical embolectomy over traditional catheter-guided thrombectomy are highlighted in this complex case of massive PE.

## Introduction

Pulmonary embolism (PE) is a very common condition worldwide with about 600,000 cases diagnosed annually in the US alone [1]. A meta-analysis of acute PE revealed that it is associated with a mortality rate of 17.4% [2]. In acute presentations of PE, prompt diagnosis and treatment are essential for patient survival and improved clinical outcomes. Computed tomography (CT) scan is the imaging modality of choice for the diagnosis of PE. The treatment of acute PE consists of cardiopulmonary support, systemic anticoagulation, and thrombolysis or reperfusion of the pulmonary vasculature [3]. Either catheter-directed embolectomy or surgical embolectomy is utilized in the treatment plan, with current trends favoring the former for routine management [4]. However, surgical embolectomy as the treatment modality of choice in an acute PE presentation has garnered intense debate. Factors such as patient presentation and hemodynamic stability determine the appropriate treatment modality [5]. In this case report, we present the surgical intervention for a patient who developed hemodynamic instability due to a clot-in-transit in a patent foramen ovale (PFO).

## Case presentation

**Figure 1:**
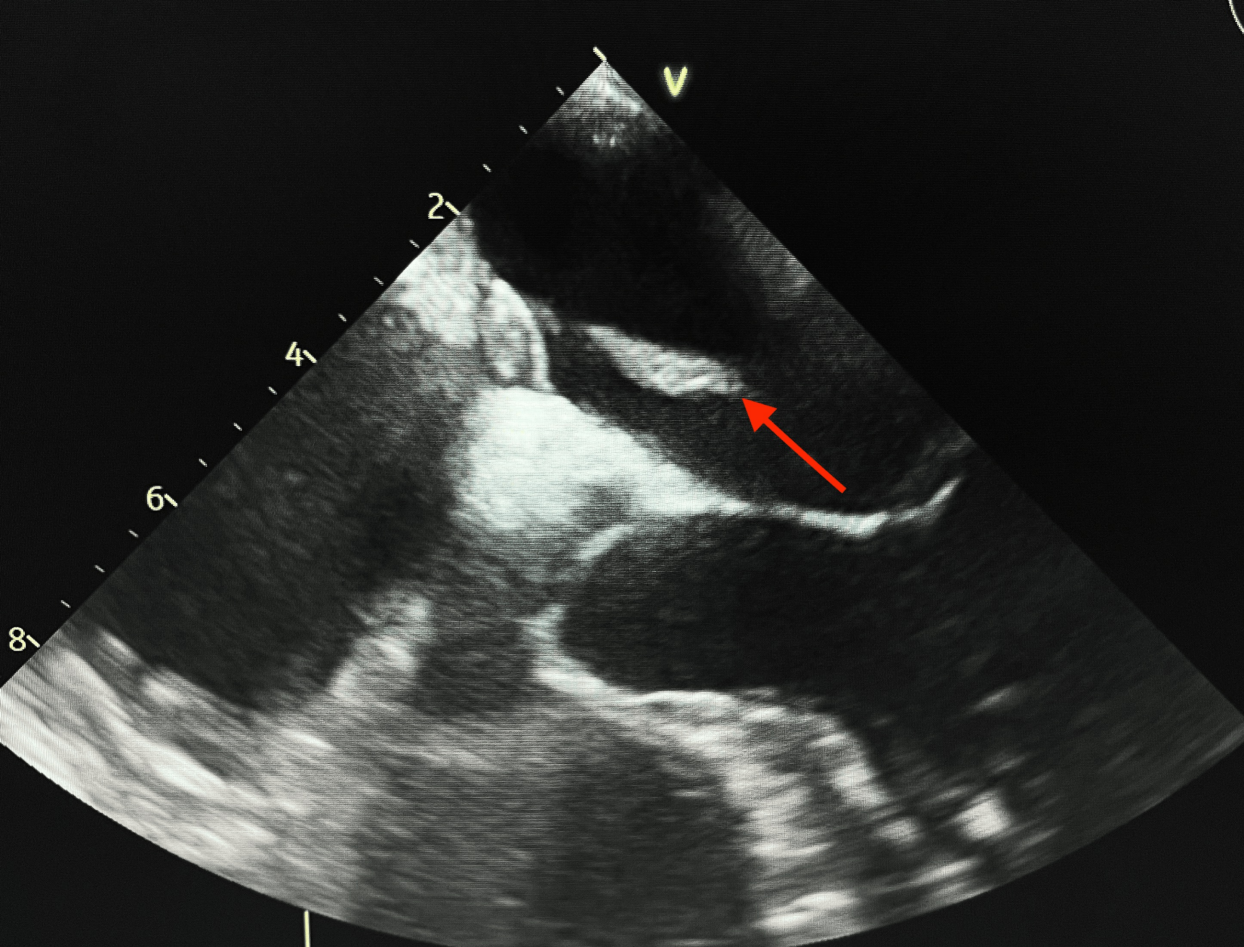
Echo demonstrating biatrial thrombus (red arrow) in the patent foramen ovale

A 30-year-old female patient arrived at the emergency department with shortness of breath, diaphoresis, tachypnea, and tachycardia. Her pertinent medical history included polycystic ovarian syndrome, oral contraceptive use, and obesity. Consequently, a CT scan of the chest was performed, which suggested a thrombus lodged in the pulmonary artery. Further lab investigation showed troponin elevation of 0.17 ng/mL (normal value: 0-0.04 ng/mL) and D-dimer >2 µg/mL (normal value: <0.4 µg/mL), therefore, cardiology was consulted for potential PE and catheter-directed thrombectomy treatment. A transthoracic echocardiogram (TTE) was performed to assess for abnormalities, revealing findings that necessitated an emergent embolectomy in the catheter lab. The echocardiogram showed a mobile echodensity extending from the atrial septum into the right atrium resulting in right heart dysfunction, which prompted concern for a PFO, as seen in the TEE (Figure 1). 

**Figure 2:**
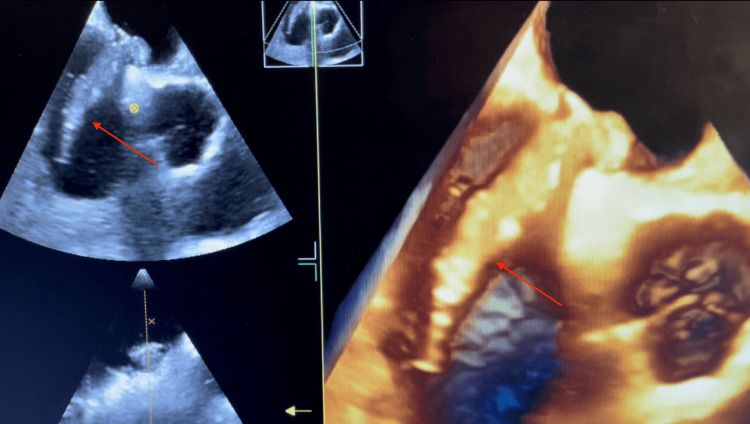
A 3-D echo demonstrating the thrombus extending from the right atrium into the patent foramen ovale (red arrows)

Cardiothoracic surgery was consulted immediately and a transesophageal echocardiogram (TEE) was recommended, which confirmed a mass extending from the atrial septum and crossing the PFO with a large portion located within the left atrium and another portion in the right atrium (Figure 2).

**Figure 3:**
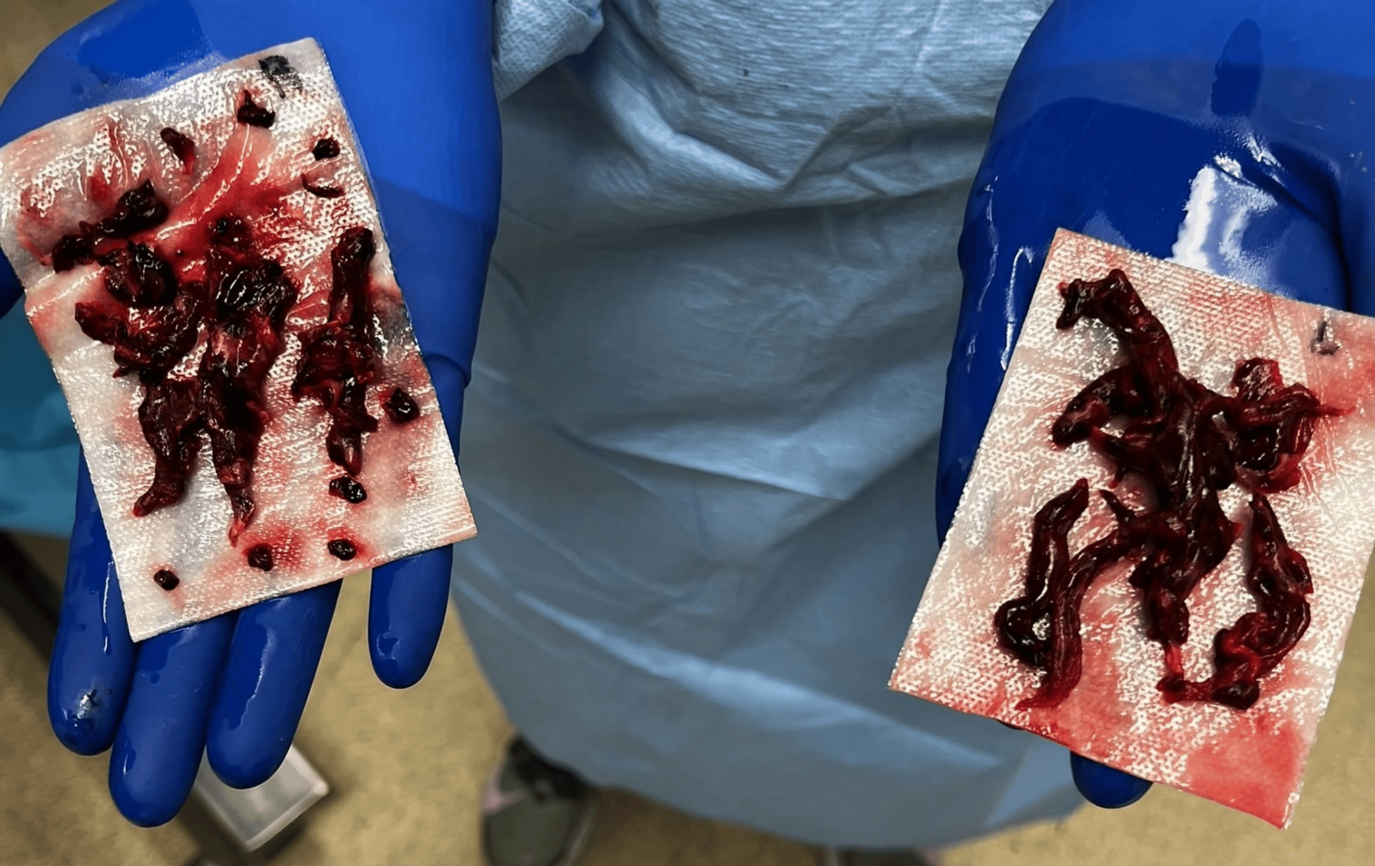
Results of the surgical pulmonary embolectomy The contents of the right pulmonary artery are shown on the left side of the figure, and the contents of the left pulmonary artery are shown on the right side.

The patient was consequently diagnosed with a submassive clot in-transit crossing the PFO into the left atrial chamber from the right one. As a result, the patient was urgently transferred to the operating room for surgical intervention as catheter-directed thrombectomy was deemed unsafe due to the location of the thrombus. Following the initiation of anesthesia, the patient began to experience hypoxia that persisted despite endotracheal intubation with 100% fraction of inspired oxygen (FiO2). An arterial blood gas confirmed a partial pressure of oxygen (PaO2) of 59% (normal: 75-100%), and a repeat TEE was performed immediately which revealed an absence of the biatrial thrombus at the interatrial septum. Severe right ventricular failure and mild-to-moderate tricuspid regurgitation was also noted with a PFO demonstrating a left to right shunt (Figure 2). The acute change in presentation and the patient’s hemodynamic compromise, representing a progression from submassive to massive PE-in-transit, prompted the decision to perform a sternotomy with emergent bilateral pulmonary embolectomy rather than typical catheter-directed procedure. Figure 3 represents the gross examination of the products of the bilateral clot removal.

The main pulmonary artery was repaired with a pericardial patch augmentation to prevent future stenosis. Additionally, primary closure of the PFO with oversewing of the atrial septum was performed. Thereafter, a bubble study was performed to confirm the closure of the foramen ovale. 

## Discussion

Acute PE is a cardiovascular condition that is associated with significant morbidity and mortality rates, including complications such as cryptogenic stroke [6]. With the rising prevalence of risk factors in patients, treating acute pulmonary emboli has become an increasingly important consideration in healthcare. In recent years, a general shift towards non-invasive procedures has led to a preference for catheter-guided thrombectomy or systemic thrombolysis instead of surgical intervention. Early opponents of surgical embolectomy were wary of the treatment due to the high in-hospital mortality rates (around 36% in the 2000s). However, following advancements in surgical technologies, the operative mortality rate has declined significantly to 4% [2]. In a nonrandomized study, patients who underwent surgical embolectomy versus thrombolysis for massive pulmonary emboli showed lower mortality rates and decreased recurrence rates of PE [7]. This case was not conducive to a typical catheter-directed procedure because of the risk of paradoxical embolism through the PFO. Therefore, early recognition of the hemodynamic instability and swift sternotomy with embolectomy was the most optimal treatment plan. Hypotension is a significant sign that the patient may require further workup for a clot-in-transit [8].

This case report was unusual in that even though a PFO exists in about a third of the population, the risk of paradoxical arterial embolism is less than 2% [9]. Because of its rare occurrence, treatment algorithms have not been substantiated or standardized. This case demonstrates that for a paradoxical PE-in-transit through a PFO causing hemodynamic collapse, surgical embolectomy is the first-line treatment to be considered for lower mortality rates. 

Upon review of the case, it was hypothesized that the biatrial thrombus was dislodged from its residence within the PFO during the induction of anesthesia in combination with positive pressure ventilation, which increased intrapulmonary pressures. Furthermore, the increased intrathoracic pressure from the ventilation altered the normal pressure gradients within the heart, consequently creating a left-to-right shunting of blood, propelling the embolus through the right atrium from the atrial septum and into the pulmonary vasculature. This prompted the patient's sudden hemodynamic compromise. Although the sudden onset of hemodynamic instability observed in this case after ventilation induction represents a strong correlation between the events, direct inferences cannot be drawn due to the uniqueness of the clinical situation. As a result, further research may be indicated for the direct cause-and-effect relationship between ventilation pressure changes and shunt reversal as evidenced by the mobilization of the clot after ventilation induction in a PFO with right to left interatrial shunt. Moreover, the specificity of this case report limits its generalizability to the broader population due to the case's complexity and the presence of confounding factors, such as the patient's past medical history and emergent circumstances. Apart from case reports, there is a lack of substantiating evidence exploring the relationship between positive pressure ventilation and shunt reversal through an interatrial septum.

## Conclusions

This case was unsuitable for a typical catheter-directed intervention because of the presence of a PFO, the location of the clot in the left atrial chamber, and the abrupt loss of hemodynamic stability. Upon reflection, a reduction of patient mortality may be achieved with surgical embolectomy for hemodynamically-unstable patients. Clinicians managing acute pulmonary emboli should consider surgical embolectomy earlier in the treatment plan instead of a salvage intervention to prevent hemodynamic collapse.

Surgical embolectomy was demonstrated to be the most appropriate treatment method for this patient. As hypothesized, the induction of ventilation contributed to an iatrogenic shunt reversal allowing the biatrial thrombus previously straddling the PFO to embolize to the pulmonary vasculature. While catheter-guided thrombectomy was initially planned with no indications for surgical embolectomy, the transition from submassive to massive PE with subsequent hemodynamic collapse highlighted the need for an adequately prepared surgical team to deal with the emerging complications. Early notification and preparation of a cardiothoracic surgical team are recommended to ensure timely intervention with emergent sternotomy and embolectomy for such patients.
